# Plant Functional Traits and Diversity of Community Link to the Plant Invasion Dominance in the Subalpine Wetland of Shennongjia National Nature Reserve, China

**DOI:** 10.3390/plants15111702

**Published:** 2026-05-31

**Authors:** Ruifeng Zhang, Hengyu Xiong, Yuanyuan Liu, Yuhan Xu, Ligai Huang, Bingnan Wen, Wenchao Zhao, Ping Zhao, Binyuan Xu, Yanfeng Bai, Ran Meng

**Affiliations:** 1 Research Institute of Forestry, Chinese Academy of Forestry, Beijing 100091, China; ruifengzhang1863@163.com; 2 Key Laboratory of Plant Genetics and Molecular Breeding, Zhoukou Normal University, Zhoukou 466001, China; 3 School of Ecology, Hainan University, Haikou 570228, China; 4 Green Intelligence Environmental School, Yangtze Normal University, Chongqing 408100, China; 5 School of Machinery and Automation, Weifang University, Weifang 261000, China; 6 College of Horticulture and Forestry Sciences, Huazhong Agricultural University, Wuhan 430070, China; 7 College of Resources and Environment, Huazhong Agricultural University, Wuhan 430070, China; 8 Faculty of Computing, Harbin Institute of Technology, Harbin 150001, China

**Keywords:** alien invasive species, Dajiuhu subalpine wetland, functional diversity, species diversity, trait–invasion linking

## Abstract

Plant invasions pose a significant threat to plant community integrity at high latitudes and altitudes, particularly under the backdrop of ongoing climate change and anthropogenic disturbance. However, how plant invasion and increasing invasion intensity reshape community functional traits and multidimensional diversity in high-altitude wetland ecosystems remain poorly understood. Here, we conducted a field survey across 284 quadrats in a subalpine wetland of Shennongjia National Nature Reserve, China. Nine invasive plant species were detected and occurred in 51.06% of all sampled quadrats. We compared functional trait composition between invaded and uninvaded communities and assessed species, functional, and phylogenetic diversity along invasion intensity gradients through inclusion and exclusion models of invasive species. Invaded communities showed 9.1% higher chlorophyll content and 30.7% larger specific leaf area but 26.1% lower leaf density than uninvaded communities. In addition, community-weighted traits and diversity indices showed stronger responses when invasive species were included. With increasing invasion intensity, species diversity and phylogenetic diversity declined, whereas functional richness increased. These results demonstrate that plant invasion simultaneously drives species loss and functional reorganization, reshaping both the functional composition and biodiversity of subalpine wetland communities. Our findings highlight how invasive species restructure plant communities in subalpine wetlands, with important implications for biodiversity conservation in high-altitude ecosystems.

## 1. Introduction

Globalization has accelerated the spread of invasive plants, enabling them to establish themselves in new regions at an unprecedented rate [1,2]. The establishment of invasive plants can substantially alter community structure and species interactions, thereby influencing the biodiversity of the community. Previous studies have shown that plant invasions may result in either an increase or a decrease in the diversity of plant communities, with the direction of this effect being largely determined by the spatial scale at which the study is conducted and by the intensity of the invasion process [3,4,5,6,7,8]. In the case of increased diversity, it is primarily due to the coexistence of invasive plants with native plant communities after invasion, and a major reason for this coexistence mechanism is the presence of functional trait differences between invasive and native communities [9,10,11]. Ecological niche differentiation theory predicts that functional trait differences between invasive and native communities reduce competitive exclusion and promote coexistence [12,13]. Conversely, as diversity declines, habitat filtering preferentially favors dominant species possessing superior trait characteristics, such as greater specific leaf area (SLA) and reduced root-to-shoot ratio, in comparison with native community flora [14,15]. This selective advantage enhances the competitive ability of these dominant species. As a result, they are able to exclude weaker competitors from the community, which in turn leads to a reduction in overall community diversity. Therefore, invasion-driven changes in diversity are highly context-dependent, varying across ecosystems and invasion intensity, regulating community stability and ecosystem functions by changing the balance between species coexistence and competitive exclusion.

Community diversity is an important determinant of ecosystem productivity, and high-diversity communities tend to resist plant invasions more effectively [16,17,18,19]. Understanding the mechanism of plant invasions on diversity is important for elucidating ecological processes and informing biodiversity management. Previous research on community diversity has focused on three dimensions: taxonomic, functional, and phylogenetic diversity. Recent studies have increasingly emphasized functional diversity relative to taxonomic and phylogenetic diversity, as ecosystem processes may be driven by plant functional diversity rather than species diversity [20,21,22,23,24]. The functional traits of plants respond dynamically to environmental variation and reflect how species cope with changing conditions [25]. When invasive plants establish in recipient communities, their functional traits exhibit differential matching with native species, thereby influencing the competitive outcomes between them. Specifically, invasive species usually exhibit trait combinations that enable them to exploit underutilized niche space. Certain invaders outperform native competitors through traits suited to novel environments, such as lower SLA or heavier seeds [26]. These trait advantages can modify community functional diversity by altering community-weighted mean trait values, thereby reshaping the dominant functional strategies and partially replacing or reordering the functional space originally occupied by native species [27]. In addition, previous studies have shown that phylogenetic diversity is more effective in predicting wetland plant community resistance to invasive plants than species richness, such as *Eichhornia crassipes* [28]. These studies on functional traits and phylogenetic relationships provide important insights into the mechanisms underlying plant invasion. However, most of these conclusions come from relatively simplified experimental systems, and most relevant studies rely on greenhouse experiments with a single invasive species. Given that plant responses to invasion differ between controlled and natural field settings and vary across spatial scales, multiple invasive species may co-occur in natural communities, leading to complex interactions between invasive and native species. These complex interactions result in changes in diversity that differ from those observed under single-species invasions. Consequently, the mechanisms identified in the laboratory may not hold in natural communities with multiple co-occurring invaders.

Community composition changes as invasion proceeds, and associated changes in diversity are often non-linear. Such patterns may arise from time lags in the establishment of invaders, feedback between invasive and native species, and threshold shifts in resource availability at different invasion intensities [29]. Consequently, the effects of invasion on biodiversity depend not only on invasion intensity but also on the identity and ecological strategies of dominant species. In addition, high-performance native dominants may buffer diversity loss by facilitating coexistence, whereas dominant invasive species can amplify biodiversity decline by monopolizing resources and excluding weaker competitors, further contributing to this non-linear pattern [30,31,32]. Such complexity has led to challenges in quantifying invasion impacts and contributed to ongoing debate over how these effects should be assessed. In particular, it remains difficult to separate the contribution of invasive species from that of the remaining native community. When diversity is evaluated at the community level, the effects of invasive species are often confounded with shifts in the native assemblage, making their individual roles difficult to isolate. However, few studies have explicitly separated these two components, leaving the mechanisms underlying diversity change poorly resolved.

To evaluate the effects of plant invasion on community biodiversity and functional structure, we conducted a field survey along a natural invasion gradient in the Dajiuhu wetland, a biodiversity-rich subalpine wetland in Shennongjia, Hubei Province, China. In recent years, this wetland has experienced increasing establishment and spread of alien invasive plants, making it an important natural system for investigating the ecological consequences of plant invasion. However, how plant invasions affect the multidimensional diversity of communities in this wetland remains unclear, limiting the understanding of invasion impacts on community assembly and biodiversity conservation in subalpine wetlands. To address this question, we compared community-weighted means (CWM) of functional traits between invaded and uninvaded communities to assess potential shifts in overall trait composition associated with plant invasion. Then, we applied paired inclusion and subtraction models to explicitly separate the contribution of dominant invasive species from that of the resident native community [7], thereby allowing a more refined evaluation of how invaders influence community-level trait structure and diversity patterns. In addition, we examined how multidimensional diversity changed systematically along the invasion intensity gradient to capture broader community responses under increasing invasion pressure. Based on previous studies, we predicted that plant invasion would lead to a decline in species, phylogenetic, and functional diversity while changing community-weighted functional traits toward more acquisitive strategies. We further predicted that these changes would be more pronounced with increasing invasion intensity and that the contribution of invasive species would drive distinct patterns of functional and diversity reorganization along the invasion gradient.

## 2. Results

### 2.1. Community Composition and Invasive Species

A total of 101 vascular plant taxa were recorded in 284 quadrats, belonging to 32 families and 73 genera. Species identified as representative dominant species in the surveyed communities included *Calamagrostis epigeios*, *Digitaria sanguinalis*, *Agrostis clavata*, *Panicum bisulcatum*, and *Elymus dahuricus* (Table S1).

Nine invasive plant species were recorded in the Dajiuhu wetland, belonging to four families and seven genera, representing 8.91% of all recorded species. Invasive plants occurred in 145 of the 284 quadrats, accounting for 51.06% of all surveyed plots, indicating that plant invasion was widespread throughout the study area. Six invasive species formed dominant communities, including *Bidens frondosa*, *Erigeron annuus*, *Coreopsis lanceolata*, *Trifolium repens*, *Trifolium pratense*, and *Oenothera glazioviana*. Three additional invasive species were recorded only occasionally, including *Erigeron canadensis*, *Lolium perenne*, and *Robinia pseudoacacia*.

### 2.2. Effect of Plant Invasion on Traits and Diversity of Plant Communities

**Figure 1:**
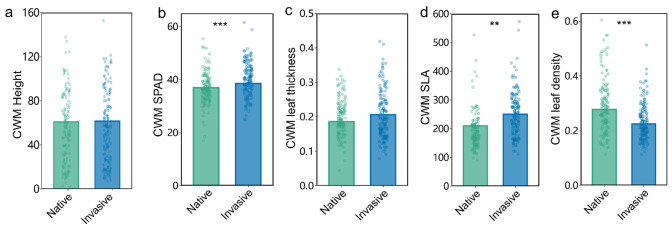
Community-weighted means (CWM) (+SE) of height, SPAD, leaf thickness, SLA, and leaf density of invasive and native plant communities in Dajiuhu wetland. (**a**) CWM of plant height, CWMHeight; (**b**) CWM of relative chlorophyll content, CWMSPAD; (**c**) CWM of leaf thickness CWMLeaf thickness; (**d**) CWM of specific leaf area, CWMSLA; (**e**) CWM of leaf density. *** *p* < 0.001, ** *p* < 0.01 (chi-square test; *n* = 145 for invasive communities and *n* = 139 for native communities).

Overall, invaded and native communities differed significantly in several functional traits, indicating shifts in community trait composition associated with plant invasion. Invasive communities had significantly higher CWM_SPAD_ (+9.1%, χ^2^ = 11.539, *p* < 0.001) and CWM_SLA_ (+30.7%, χ^2^ = 7.431, *p* < 0.01) but lower CWM_Leaf density_ (−26.15%, χ^2^ = 11.158, *p* < 0.001) than native communities (Figure 1b,d,e; Table 1). Height and leaf thickness showed no significant differences between invasive and native communities (Figure 1a,c; Table 1).

**Figure 2:**
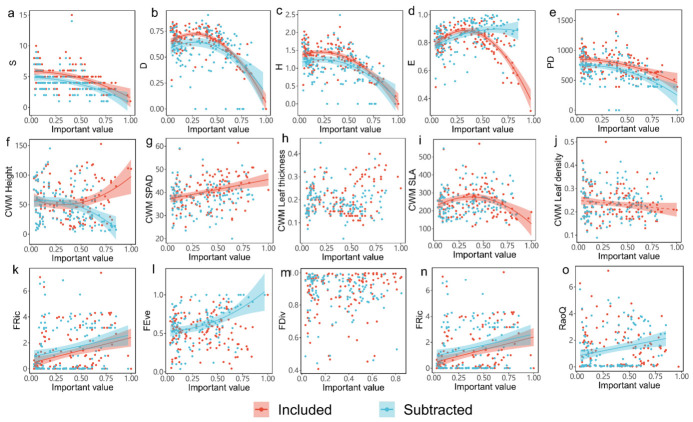
Relationship between the important value of invasive species and the diversity of herbaceous plant communities. (**a**) Gleason species richness, S; (**b**) Simpson’s diversity index, D; (**c**) Shannon–Weiner’s diversity index, H; (**d**) Pielou’s evenness index, E; (**e**) phylogenetic diversity, PD; (**f**) community-weighted means (CWM) of plant height, CWMHeight; (**g**) CWM of relative chlorophyll content, CWMSPAD; (**h**) CWM of leaf thickness CWMLeaf thickness; (**i**) CWM of specific leaf area, CWMSLA; (**j**) CWM of leaf density, CWMLeaf density; (**k**) functional richness, FRic; (**l**) functional evenness, FEve; (**m**) functional divergence, FDiv; (**n**) functional dispersion, FDis; (**o**) Rao’s quadratic index, RaoQ. Blue data points and trend lines indicate values and models for the resident communities (i.e., subtraction models). Pink data points and trend lines indicate values and models for the resultant communities (i.e., inclusion models). Solid lines indicate model significance, *p* < 0.05; dashed lines indicate model significance, 0.05 < *p* < 0.1. Trends depicted represent the fixed effect component of linear mixed-effect models.

Invasive species shaped community diversity and functional traits along the invasion gradient (Figure 2; Table S2). By contrasting statistical models that included or subtracted invasive species, we isolated the effect of the invader on community diversity and traits. While several indices (S, D, H, PD, and Fric) showed consistent responses in both models (Figure 2a–c,e,k), other indices exhibited different performances. In particular, E increased continuously with the importance value of invasive species in the subtracted model but displayed a unimodal response in the included model (Figure 2d). In contrast, CWM_Height_ exhibited a U-shaped pattern in the included model but declined sharply in the excluded model (Figure 2f).

Other community-weighted traits showed no significant relationships in subtracted models, whereas included models showed higher CWM_SPAD_, a non-linear response of CWM_SLA_, and lower CWM_Leaf density_ with the increasing importance value of invasive species (Figure 2g,i,j). Functional diversity indices (FEve, FDis, and RaoQ) increased gradually with the importance value of invasive species in subtracted models but showed no clear patterns in included models. Additionally, CWM_Leaf thickness_ and FDiv were both unpredictable in two models (Figure 2h,m).

### 2.3. Differences in Community Trait Values and Diversity Across Different Invasion Intensities

**Figure 3:**
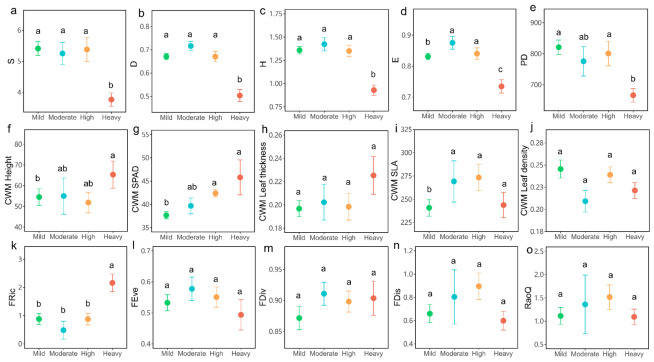
Differences in community trait values and plant diversity across different levels of invasion intensity (±SE). Mild: invasive species relative coverage ≤ 25%; moderate: 26% ≤ invasive species relative coverage ≤ 50%; high: 51% ≤ invasive species relative coverage ≤ 75%; heavy: invasive species relative coverage ≥ 76%. Different letters indicate significantly different EMMeans. The meaning of all indicators in each subfigure is identical to that described in Figure 2. *n* = 145 for all invasive communities.

Diversity and traits varied significantly among invasion intensity levels. As the degree of invasion increases, species diversity (S, D, H, E) and phylogenetic diversity (PD) remain stable initially but significantly decrease when invasion reaches the heavy degree (Figure 3a–e; Table S3), while CWM_Height_, CWM_SPAD_, Fric, and CWM_SLA_ significantly increase at the heavy invasion degree (Figure 3f,g,k,i; Table S3). However, CWM_leaf thickness_ and CWM_leaf density_, as well as functional diversity indices (FEve, FDiv, FDis, and RaoQ), showed no significant differences among different invasion degrees (Figure 3h,j,l–o; Table S3).

## 3. Discussion

We found that plant invasion changed community functional traits, characterized by increased CWM_SPAD_ and CWM_SLA_ and decreased CWM_Leaf density_. To disentangle the contribution of invasive species, we compared models including versus subtracting invaders in invaded communities. While basic species diversity indices showed largely consistent patterns between the two models, traits and functional diversity appeared different. The paired inclusion–subtraction approach provided a method for distinguishing the direct contribution of invasive species from the response of the remaining native community. Further, we examined how these indices varied along with invasion intensity. Heavily invaded communities showed reduced species diversity and phylogenetic diversity, but increased CWM_Height_ and functional richness (FRic). Our findings improved understanding that plant invasion could drive species loss and functional reorganization simultaneously during community assembly.

### 3.1. Plant Invasion Drove Changes in Community Trait Composition and Diversity

The competitive advantage of invasive species is often rooted in superior functional traits, such as larger leaf area, higher photosynthetic rates, greater height, and higher biomass allocation relative to native species [33,34,35]. Consistent with this widely recognized pattern, we found that the establishment and increasing dominance of invasive species were associated with changes in community-level trait composition (Figure 1). Invaded communities showed significantly higher chlorophyll content and larger SLA, both of which are traits closely associated with enhanced productivity, faster resource acquisition, and stronger competitive vigor [15,36,37,38]. The changes in traits indicated a tendency toward more acquisitive strategies in invaded communities, which facilitated rapid growth and competitive dominance under favorable conditions. However, the leaf density of invaded communities was significantly lower than that of uninvaded communities, suggesting a relatively reduced level of structural robustness and potentially a lower capacity for stress tolerance when compared to native-dominated communities. This pattern might be related to the absence or reduction in natural enemies in invaded habitats, which decreased the biotic pressure on invasive species [39,40,41]. As a result, invasive species could allocate more resources to growth and reproduction rather than to defensive traits. The changes in traits suggested that plant invasion altered the dominant ecological strategies of the community. By favoring species with rapid resource acquisition, plant invasion may influence community succession and ecosystem function through competitive interactions and resource strategies.

Regression analysis revealed that, in addition to causing significant shifts in community-weighted means, species diversity and phylogenetic diversity were both significantly negatively correlated with the dominance of invasive plants (Figure 2). The results were consistent with the study by Livingstone et al. [7]. As the level of invasiveness exhibited by plant species increased, both the species and phylogenetic diversity of communities declined, further indicating the stronger competitive abilities of invasive plants compared with native species. The persistent and consistent impact may potentially disrupt the stability and resilience of regional ecosystems over time. In contrast to previous research findings [42,43], the high functional richness of the community was closely associated with greater invasiveness in this study. The results may be due to the leaf traits, which differ markedly between invasive and native species and may expand, rather than compress, community functional space. Consequently, the expansion of functional space may enhance resource use efficiency and contribute to productivity. The consistency between trait changes and diversity responses suggests that invasive species alter both the functional composition and phylogenetic structure of the community.

### 3.2. Heavy Invasion Restructures Community Diversity and Functional Traits

As invasive species dominance increased, chlorophyll content and height at the community level increased significantly, potentially indicating enhanced competitive ability and productivity [44]. In contrast, leaf density decreased, and SLA showed a non-linear response to increasing invader dominance, indicating a more complex, potentially opposing pattern. These results highlighted that changes in functional traits do not always mirror changes in growth or biomass. In addition, subtraction models revealed that both Pielou’s evenness (E) and functional evenness (FEve) increased as invasive species became more dominant (Figure 2). They indicated that the remaining native species were more evenly distributed in both abundance and functional trait space when removing the contribution of invasive species was removed. Along with a decline in diversity in communities, including invasive species, this pattern highlighted invasion-driven homogenization [45,46,47,48]. However, some studies suggested that community homogenization driven by plant invasion was not absolute, depending on the invasion stage, as well as the life history traits and growth forms of the invasive species [49]. Therefore, invasive species played a key role in shaping community diversity and functional structure, and this process was modulated by traits and strategies of invasive species.

Plant invasions typically occur via a gradual process of population establishment, and this process, in turn, alters the course of community succession. The different levels of invasion intensity that we recorded in this study mirrored this gradual progression, and they brought about detectable effects on community structure as well as diversity. Specifically, the indices of species diversity did not change appreciably under conditions of low or moderate invasion. The species diversity indices remained stable under low and moderate invasion levels but decreased significantly under heavy invasion (Figure 3), which is consistent with previous studies [11,50,51,52]. The results indicated that relatively low levels of invasion did not necessarily enhance overall species diversity, possibly due to the comparatively lower competitive ability or less pronounced allelopathic effects of invasive species in effectively excluding other native species within the community. Conversely, heavier invasion levels, accompanied by stronger allelopathic effects, will significantly undermine the community [53]. More importantly, we found that functional richness remained unchanged across the invasion gradient but increased significantly under heavy invasion (Figure 3k). This result contrasted with previous studies and our hypothesis, reducing or unchanging in functional diversity under plant invasion [29,54], and suggested that severe invasion could expand community trait space by introducing novel trait combinations rather than uniformly constraining functional composition. The increasing functional richness, together with the higher chlorophyll content and plant height under heavy invasion, suggested that invasive species possessed trait combinations enhancing resource acquisition and competitive dominance under high invasion pressure. Differences in functional traits among invasive species may potentially explain the considerable variation in their relative invasiveness, and the key traits of dominant species usually play a critical role in shaping the functional composition of the community. Studies have demonstrated that invasive species with higher photosynthetic capacity may gain a competitive advantage over native species by capturing resources more efficiently and sustaining faster growth under favorable conditions [15,55]. This mechanism was also supported by the observed increases in community chlorophyll content and specific leaf area. Meanwhile, competitive superiority derived from increased plant height allows certain invasive species to directly suppress neighboring plants via more efficient light competition [43]. The interpretation was consistent with the significant increase in community-weighted mean height along the invasion gradient. In addition, invasive plants usually have high propagule pressure and clonal reproductive capacity. The clonal growth strategy enabled invasive plants to rapidly propagate and form monoculture dominance in heterogeneous environments [56], such as the invasion of *C. lanceolata* by clonal propagation in sunny habitats [57,58]. The coexistence of invasive species with contrasting functional traits may allow them to occupy different portions of community trait space. This mechanism may partly explain the increase in functional richness observed under heavy invasion. Given that different invasive species may rely on distinct trait combinations and ecological strategies, future studies in this wetland should compare the traits of invasive and co-occurring native species. These comparisons would help identify species-specific mechanisms of invasiveness and clarify how different invaders contribute to community reassembly.

It should be noted that this study did not distinguish the heterogeneous contributions of different invasive species, and, therefore, the above discussion on trait differences was mainly based on the existing literature rather than direct evidence from this study. Nevertheless, our analysis still revealed that plant invasion can substantially reshape community functional structure and multiple dimensions of biodiversity in a subalpine wetland ecosystem, changing overall community trait strategies toward higher resource acquisition. Species diversity and phylogenetic diversity decrease along the invasion gradient, but functional richness increases with invasion intensity, suggesting that invasion expanded rather than constrained community trait space. These highlighted the complex effects of invasive species on community assembly, where species loss and functional reorganization occurred simultaneously. Although our results provided valuable insights into how invasion affects multidimensional biodiversity, fully disentangling the underlying causal mechanisms remains challenging. Moreover, our analysis was based on a single temporal snapshot, which could not capture the dynamic changes or long-term ecological feedbacks associated with the invasion process. Therefore, future studies in this field need to integrate multiple complementary approaches, including multi-species comparisons, long-term monitoring efforts, and experimental manipulations, in order to comprehensively elucidate both the underlying mechanisms and the broader ecological consequences of invasion-driven community reassembly.

## 4. Materials and Methods

### 4.1. Study Sites

**Figure 4:**
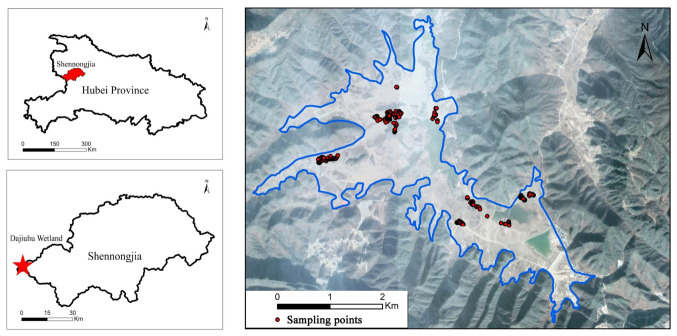
Geographical location and survey sampling points distribution in Dajiuhu subalpine wetland of Shennnongjia National Nature Reserve, China. The red circles are the coordinates of each quadrat. The upper-left panel shows a map of Hubei Province, China. The lower-left panel indicates the location of the Shennongjia Nature Reserve within Hubei Province, with the red star marking the sampling site at Dajiuhu Wetland. The right panel shows the distribution of quadrat within the Dajiuhu Wetland.

The study area was in the Dajiuhu National Wetland Park, a subalpine wetland of Shennongjia National Nature Reserve, China (109°56′~110°11′ E, 31°34′~31°36′ N) (Figure 4). This area is located at an average elevation of approximately 1730 m and experiences a subalpine cold temperate and humid climate, with an average annual temperature of 7.4 °C and precipitation of 1528.3 mm [59,60]. There are several typical subalpine wetlands in the park, such as subalpine meadows, *Sphagnum palustre* peat bogs, and marshes of *C. epigeios* or *Typha angustifolia*. Invasive plants have been discovered since 1976, and more than 20 species have been identified to date [61].

### 4.2. Sampling Design and Data Collection

In August 2023, seven survey sites were selected in the study area. These sites were chosen specifically to represent the major types of herbaceous communities present in the region. To avoid spatial autocorrelation, a minimum distance of 300 m was maintained between any two neighboring sites. Within each selected site, a stratified sampling design was applied, which was based on the percentage cover of dominant species. Under this design, multiple 1 m × 1 m quadrats were established within representative herbaceous communities. In total, 284 quadrats were surveyed, and the spatial distribution of these quadrats is presented in Figure 4. All plant species occurring within each quadrat were identified either directly in the field or subsequently in the laboratory. Species identifications were verified using the Flora of Shennongjia [62] and classified as native or invasive alien to China based on the Flora of China (www.efloras.org accessed on 16 August 2023), Invasive Alien Species of China (IASC) (www.iplant.cn/ias accessed on 16 August 2023) [63,64]. The cover of each species was estimated visually, and all estimates were made by a single observer to ensure consistency across quadrats. The precision of these cover estimates was 1%. The surveys were carried out during the peak of the growing season, a time when the majority of plant species were at a stage that allowed for reliable identification.

We selected plant height, leaf relative chlorophyll content (SPAD), leaf thickness, specific leaf area (SLA), and leaf density as key functional traits. They capture ecological strategies of species, such as competitive ability, resource acquisition, and stress tolerance, and collectively reflect a broad range of ecosystem functions [65,66,67]. Plant height and SPAD of the leaf were measured in the field using a tape measure and a SPAD-502 chlorophyll meter (Konica Minolta Sensing, Osaka, Japan), respectively. Mature leaf samples were collected, transported to the laboratory in an icebox, and immediately used to measure other functional traits. Leaf thickness was measured using an electronic vernier caliper with a precision of 0.01 mm. SLA was calculated as the ratio of leaf area to leaf dry weight. Leaf area was quantified using ImageJ 1.53, and leaf dry weight was weighed after drying at 80 °C for 72 h. Leaf density was calculated as leaf dry weight divided by leaf volume.

### 4.3. Assessment of Species Diversity

The calculation of species diversity indices was based on important values and performed using the Vegan package implemented in R version 4.0.2. These diversity indices included species richness (S), the Simpson index (D), the Shannon–Weiner index (H), and Pielou’s evenness index (E) [68,69]. Important values, which served as a measure of invasion intensity, were computed as the average of relative cover and relative height. The species diversity indices were then calculated according to the following formulas:

$$D=1-\sum_{i=1}^{s}P_{i}^{2}$$

$$H=-\sum_{i=1}^{S}P_{i}lnP_{i}$$

$$E=\frac{-\sum_{i=1}^{S}P_{i}lnP_{i}}{lnS}$$

where *S* is the number of plant diversity in the community and *Pi* represents the proportion of individuals of species *i* relative to the total [70,71].

### 4.4. Assessment of Functional Diversity and Phylogenetic Diversity

The functional diversity indices (including FRic, FEve, FDiv, FDis, RaoQ) and community-weighted mean traits (CWM_Height_, CWM_SPAD_, CWM_Leaf thickness_, CWM_SLA_, CWM_Leaf density_) were calculated using the FD package in R version 4.0.2 [72]. The functional traits used to calculate these indices are plant height, SPAD, leaf thickness, SLA, and leaf density. Phylogenetic diversity (PD) for these plots was calculated using the ape [73] and picante packages [69]. Functional diversity indices were calculated as follows:

$$FRic=\frac{SF_{ic}}{R_{c}}$$

where *SF_ic_* is the size of the functional trait space occupied by species within community *i* and *Rc* is the absolute range of trait *c*.

$$EW_{i}=\frac{dist\left(i,j\right)}{w_{i}+w_{j}}$$

$$PEW_{i}=\frac{{EW}_{i}}{\sum_{l=1}^{s-1}EW_{i}}$$

$$FEve=\frac{\Sigma \min\left(PEw_{i}\times \frac{1}{s-1}\right)-\frac{1}{s-1}}{1-\frac{1}{s-1}}$$

where *EW* is evenness, *dist*(*i*, *j*) is the Euclidean distance between two species, and *wi* is the relative abundance of species *i*.

$$FDiv=\frac{\Delta d+\bar{dG}}{\Delta \left|d\right|+dG}$$

$$\Delta |d|=\sum_{i=1}^{s}w_{i}\times \left|dG_{i}-\bar{dG}\right|$$

$$\Delta d=\sum_{i=1}^{s}W_{i}\times \left(dG_{i}-\bar{dG}\right)$$

$$\bar{dG}=\frac{1}{s}\sum_{1}^{s}dG_{i}$$

$$dG_{i}=\sqrt{\sum_{k=1}^{T}(x_{ik}-g_{k}{)}^{2}}$$

$$g_{k}=\frac{1}{S}\sum_{i=1}^{s}x_{ik}$$

where *xik* is the value of trait *k* for species *i*, *gk* is the centroid of trait *k*, *T* is the number of traits, *dGi* is the average distance of species *i* to the centroid, *d* is the abundance-weighted dispersion, and *wi* is the relative abundance of species *i*.

$$c=\left[c_{i}\right]=\frac{\sum w_{j}x_{ik}}{\sum w_{j}}$$

$$FDis=\frac{\sum w_{j}z_{j}}{\sum w_{j}}$$

where *c* is the weighted centroid, *wj* is the relative abundance of species *j*, and *zj* is the weighted distance from species *j* to the centroid *c*.

$$RaoQ=\sum_{i=1}^{S-1}\sum_{j=i+1}^{S}d_{ij}p_{i}p_{j}$$

$$d_{ij}=\frac{1}{n}\sum_{k=1}^{n}{\left(X_{ik}-X_{jk}\right)}^{2}$$

where *dij* is the difference in functional trait values between species *i* and *j*; *pi* (*pj*) is the percentage of individuals of species *i* (*j*) in the community; *S* is species richness; *n* is trait number; and *Xik* and *Xjk* are trait values of species *i* and *j*.

$$CWM=\sum_{i=1}^{n}p_{i}\times trait_{i}$$

where *n* is the number of species, *pi* is the relative abundance of species *i* in the plot, and *trait_i_* is the trait value of species *i*.

### 4.5. Statistical Analysis

To determine the functional position occupied by invasive plants within the community, we applied chi-square tests to compare community-weighted mean trait values between invaded communities and uninvaded (native) communities. The response variables in the model were CWM_Height_, CWM_SPAD_, CWM_Leaf thickness_, CWM_SLA_, and CWM_Leaf density_. In order to account for potential differences among the various communities, the identity of the dominant species in each community was included as a random factor in all of the fitted models.

In order to quantify the contributions of invasive plants to community diversity and functional traits, and to test whether plant invasion alters the relationships between these indices and the invaders’ importance values, we compared two models (inclusion and subtraction) using the lme4 package in R version 4.0.2 [56,74]. In the inclusion models, both the abundance data and the trait data of all invasive species were retained. By contrast, in the subtraction models, these same data were removed entirely. For each sample plot, after removing the abundance and trait values of the main invasive species within the subtraction model framework, we recalculated all of the diversity metrics. This step allowed us to separate two components: the effects that invaders exert on the resident native community versus the contribution of invaders themselves to the overall community diversity and trait composition. For each of the two models, we fitted both linear mixed models and polynomial mixed models. We then selected the model form that provided a better fit to the data, with the selection based on the Akaike Information Criterion (AIC), so as to balance goodness of fit with model complexity, and the statistical significance was set at a threshold of *p* < 0.05.

Further, to investigate how invasion influences both community diversity and community traits, we classified the relative coverage of invasive species within invaded communities into four distinct levels. Specifically, a relative coverage of 25% or less was defined as mild invasion; coverage falling between 26% and 50% was defined as moderate invasion; coverage between 51% and 75% was defined as high invasion; and coverage exceeding 75% was defined as heavy invasion. Subsequently, we performed Tukey’s post hoc tests to compare the differences in community biodiversity and community trait values across these four categories of invasion extent.

## 5. Conclusions

This study revealed the ecological impacts of plant invasions on the Dajiuhu subalpine wetland. Invasive plants substantially altered community functional traits, driving a shift toward trait strategies associated with high resource acquisition and competitive dominance. Both invasion intensity and invader abundance were strong predictors of community diversity. Given that different invasive species may achieve dominance via distinct functional pathways, future research should integrate individual-level trait measurements with environmental and phylogenetic data to fully elucidate the mechanisms underlying invasion success in high-altitude wetland ecosystems.

## Figures and Tables

**Table 1:** The difference in CWM traits between invasive and native communities. Values are in bold when *p* < 0.05.

	CWM_Height_		CWM_SPAD_		CWM_Leaf thickness_		CWM_SLA_		CWM_Leaf density_	
Fixed effects	χ^2^	*p*	χ^2^	*p*	χ^2^	*p*	χ^2^	*p*	χ^2^	*p*
Invasive	0.600	0.439	**11.539**	**<0.001**	1.053	0.305	**7.431**	**0.006**	**11.158**	**<0.001**
Random effects	SD		SD		SD		SD		SD	
Species	1.766		0.02		0.051		0.174		0.178	
Residual	0.874		0.225		0.061		0.23		0.247	

## Data Availability

The original contributions presented in this study are included in the article material. Further inquiries can be directed to the corresponding author.

## Supplementary Materials

The following supporting information can be downloaded at: https://www.mdpi.com/article/10.3390/plants15111702/s1, Table S1: List of dominant plant species recorded in the surveyed communities of the Dajiuhu wetland; Table S2: Linear mixed effects models for the relationship between both regional plant community diversity and community trait values and invasive species important values; Table S3: Results of linear mixed models for different degrees of invasive intensity.
