# Supplementary material for: Plant Functional Traits and Diversity of Community Link to the Plant Invasion Dominance in the Subalpine Wetland of Shennongjia National Nature Reserve, China
Source: Plants (Basel). 2026 May 31;15(11):1702. doi: 10.3390/plants15111702 (PMC13259496; doi:10.3390/plants15111702)
Supplement: Supplementary file 1 [file plants-15-01702-s001.zip › plants-4281223-supplementary.pdf]

Supporting Information

Table S1: List of dominant plant species recorded in the surveyed communities of the Dajiuhu wetland, including taxonomic information.

Family	Genus	Species	Family	Genus	Species
Lamiaceae	*Galeopsis*	*Galeopsis bifida*	Asteraceae	*Inula*	*Inula hupehensis*
Juncaceae	*Juncus*	*Juncus effusus*	Asteraceae	*Erigeron*	*Erigeron annuus*
Leguminosae	*Trifolium*	*Trifolium pratense*	Melanthiaceae	*Veratrum*	*Veratrum grandiflorum*
Leguminosae	*Trifolium*	*Trifolium repens*	Polygonaceae	*Rumex*	*Rumex acetosa*
Leguminosae	*Vicia*	*Vicia sativa*	Polygonaceae	*Rumex*	*Rumex acetosella*
Leguminosae	*Vicia*	*Vicia cracca*	Polygonaceae	*Persicaria*	*Persicaria lapathifolia*
Poaceae	*Calamagrostis*	*Calamagrostis epigeios*	Polygonaceae	*Persicaria*	*Persicaria vivipara*
Poaceae	*Digitaria*	*Digitaria sanguinalis*	Polygonaceae	*Persicaria*	*Persicaria sagittata*
Poaceae	*Agrostis*	*Agrostis clavata*	Polygonaceae	*Bistorta*	*Bistorta macrophylla*
Poaceae	*Panicum*	*Panicum bisulcatum*	Polygonaceae	*Persicaria*	*Persicaria nepalensis*
Poaceae	*Elymus*	*Elymus dahuricus*	Onagraceae	*Oenothera*	*Oenothera glazioviana*
Poaceae	*Brachypodium*	*Brachypodium sylvaticum*	Geraniaceae	*Geranium*	*Geranium rosthornii*
Poaceae	*Poa*	*Poa pratensis*	Geraniaceae	*Geranium*	*Geranium dahuricum*
Poaceae	*Poa*	*Poa annua*	Equisetaceae	*Equisetum*	*Equisetum hyemale*
Polytrichaceae	*Polytrichum*	*Polytrichum commune*	Sphagnaceae	*Sphagnum*	*Sphagnum palustre*
	Hypericum	*Hypericum monogynum*	Rosaceae	*Fragaria*	*Fragaria orientalis*
Asteraceae	*Coreopsis*	*Coreopsis lanceolata*	Rosaceae	*Sanguisorba*	*Sanguisorba officinalis*
Asteraceae	*Artemisia*	*Artemisia lavandulifolia*	Rosaceae	*Potentilla*	*Potentilla kleiniana*
Asteraceae	*Pseudognaphalium*	*Pseudognaphalium affine*	Apiaceae	*Daucus*	*Daucus carota*
Asteraceae	*Bidens*	*Bidens frondosa*	Acoraceae	*Acorus*	*Acorus calamus*
Asteraceae	*Synurus*	*Synurus deltoides*	Osmundaceae	*Osmundastrum*	*Osmundastrum cinnamomeum*

Table S2: Linear mixed effects models for the relationship between both regional plant community diversity and community trait values and invasive species important values. Model type “poly” indicates a polynomial model.

Diversity Index	Model	Type	R^2	Df	Pr(>F)	Fixed effects			
						Estimate	SE	Estimate 2	SE
S	inclusion	poly	0.229	141	<0.001	0.054	1.987	-4.307	2.337
	subtraction	poly	0.205	141	<0.001	-0.389	2.117	-3.738	2.564
SP	inclusion	poly	0.622	142	<0.001	0.680	1.050	-1.248	0.123
	subtraction	poly	0.155	141	<0.001	0.520	0.225	-0.942	0.273
SW	inclusion	poly	0.475	142	<0.001	0.986	0.323	-2.280	0.380
	subtraction	poly	0.185	141	<0.001	0.867	0.514	-1.922	0.622
P	inclusion	poly	0.494	141	<0.001	0.784	0.099	-1.197	0.120
	subtraction	poly	0.166	133	<0.001	0.410	0.134	-0.315	0.169
Fric	inclusion	linear	0.093	143	<0.001	0.437	0.225	1.967	0.512
	subtraction	linear	0.056	141	0.004	0.906	0.256	1.724	0.596
FEve	inclusion	linear	0.000	135	0.830	-0.016	0.074		
	subtraction	poly	0.171	119	<0.001	0.010	0.298	0.530	0.377
Fdiv	inclusion	linear	0.014	134	0.173	0.064	0.047		
	subtraction	linear	0.017	113	0.169	-0.072	0.052		
Fdis	subtraction	poly	0.004	141	0.739	0.567	0.798	-0.744	0.966
	subtraction	linear	0.088	134	<0.001	0.842	0.233		
RaoQ	inclusion	linear	0.000	142	0.901	-0.062	0.502	1.000	
	subtraction	linear	0.061	134	0.004	1.545	0.520		
CWMH	inclusion	poly	0.054	142	0.018	-63.293	38.214	100.926	44.246
	subtraction	poly	0.132	134	<0.001	31.817	40.156	-98.005	50.693
CWMRCC	inclusion	linear	0.112	142	<0.001	8.610	2.033		
	subtraction	linear	0.134	135	0.176	2.986	2.196		
CWMLT	subtraction	poly	0.026	134	0.164	-7.923	8.398	14.264	10.601
	subtraction	linear	0.006	135	0.387	-0.019	1.000		
CWMSLA	subtraction	poly	0.090	141	0.001	284.230	89.670	-380.770	105.500
	subtraction	linear	0.016	134	0.139	37.980	25.520		
CWMLD	inclusion	linear	0.023	142	0.071	-0.040	0.022		
	subtraction	linear	0.000	133	0.885	-0.004	0.245		
PD	inclusion	poly	0.181	142	<0.001	-11.230	212.100	-380.340	249.370
	subtraction	poly	0.203	142	<0.001	136.240	237.840	-625.290	279.620

Table S3: Results of linear mixed models for different degrees of invasive intensity. (Mild: invasive species cover ⩽ 25%; Moderate: 26% ⩽ invasive species cover ⩽ 50%; High: 51% ⩽ invasive species cover ⩽ 75%; Heavy: invasive species cover ⩾ 76%). Values are bold when *P*<0.05, and italic when 0.05<*P*<0.1.

Contrast	Diversity Index	T	*P*	Contrast	Diversity Index	T	*P*
Heavy-High	S	-3.744	**0.002**	Heavy-High	D	-5.952	**<0.001**
Heavy-Mild	S	-4.063	**<0.001**	Heavy-Mild	D	-5.806	**<0.001**
Heavy-Moderate	S	-2.902	**0.022**	Heavy-Moderate	D	-5.669	**<0.001**
High-Mild	S	-0.044	1	High-Mild	D	1.048	0.715
High-Moderate	S	-0.232	0.996	High-Moderate	D	-0.967	0.763
Mild-Moderate	S	-0.214	0.997	Mild-Moderate	D	-1.803	0.264
Heavy-High	H	-4.868	**< 0.001**	Heavy-High	E	-4.684	**<0.001**
Heavy-Mild	H	-4.938	**< 0.001**	Heavy-Mild	E	-2.803	**0.029**
Heavy-Moderate	H	-4.213	0	Heavy-Moderate	E	-4.379	**< 0.001**
High-Mild	H	0.288	0.992	High-Mild	E	2.68	**0.041**
High-Moderate	H	-0.648	0.916	High-Moderate	E	-0.565	0.942
Mild-Moderate	H	-0.876	0.813	Mild-Moderate	E	-2.649	**0.044**
Heavy-High	FRic	3.641	**0.002**	High-Mild	FEve	0.305	0.99
Heavy-Mild	FRic	4.298	**< 0.001**	High-Moderate	FEve	-0.462	0.967
Heavy-Moderate	FRic	3.794	**0.001**	Mild-Moderate	FEve	-0.701	0.896
High-Mild	FRic	0.263	0.994	Heavy-High	FDiv	0.096	1
High-Moderate	FRic	1.155	0.656	Heavy-Mild	FDiv	2.066	0.17
Mild-Moderate	FRic	1.039	0.727	Heavy-Moderate	FDiv	2.469	*0.07*
Heavy-High	FEve	-1.66	0.349	High-Mild	FDiv	2.011	0.19
Heavy-Mild	FEve	-1.514	0.432	High-Moderate	FDiv	2.425	*0.078*
Heavy-Moderate	FEve	-1.702	0.327	Mild-Moderate	FDiv	0.307	0.99

Heavy-High	FDis	-1.851	0.256	Heavy-High	RaoQ	-0.422	0.975
Heavy-Mild	FDis	-0.358	0.984	Heavy-Mild	RaoQ	0.617	0.926
Heavy-Moderate	FDis	-0.794	0.857	Heavy-Moderate	RaoQ	0.418	0.975
High-Mild	FDis	1.755	0.3	High-Mild	RaoQ	1.125	0.675
High-Moderate	FDis	0.568	0.942	High-Moderate	RaoQ	0.738	0.882
Mild-Moderate	FDis	-0.598	0.932	Mild-Moderate	RaoQ	0.011	1
Heavy-High	CWM Height	1.674	0.342	Heavy-High	CWM SPAD	0.15	0.999
Heavy-Mild	CWM Height	3.393	**0.005**	Heavy-Mild	CWM SPAD	4.651	**<0.001**
Heavy-Moderate	CWM Height	1.442	0.476	Heavy-Moderate	CWM SPAD	1.585	0.391
High-Mild	CWM Height	1.608	0.378	High-Mild	CWM SPAD	5.349	**<0.001**
High-Moderate	CWM Height	0.189	0.998	High-Moderate	CWM SPAD	1.549	0.411
Mild-Moderate	CWM Height	-0.958	0.774	Mild-Moderate	CWM SPAD	-1.588	0.389
Heavy-High	CWM Leaf thickness	-0.394	0.979	Heavy-High	CWM SLA	-0.253	0.994
Heavy-Mild	CWM Leaf thickness	-2.013	0.188	Heavy-Mild	CWM SLA	3.941	**<0.001**
Heavy-Moderate	CWM Leaf thickness	-0.723	0.888	Heavy-Moderate	CWM SLA	-0.433	0.973
High-Mild	CWM Leaf thickness	-1.747	0.304	High-Mild	CWM SLA	4.516	**<0.001**
High-Moderate	CWM Leaf thickness	-0.443	0.971	High-Moderate	CWM SLA	-0.246	0.995
Mild-Moderate	CWM Leaf thickness	0.721	0.889	Mild-Moderate	CWM SLA	-3.5	**0.004**
Heavy-High	CWM Leaf density	-1.394	0.505	Heavy-High	PD	-2.633	**0.046**
Heavy-Mild	CWM Leaf density	-0.542	0.949	Heavy-Mild	PD	-2.923	**0.021**

Heavy-Moderate	CWM Leaf density	0.87	0.821	Heavy-Moderate	PD	-1.687	0.335
High-Mild	CWM Leaf density	0.924	0.792	High-Mild	PD	-0.118	0.999
High-Moderate	CWM Leaf density	1.9	0.233	High-Moderate	PD	0.251	0.994
Mild-Moderate	CWM Leaf density	1.308	0.56	Mild-Moderate	PD	0.343	0.986
